# Exploring the Effects of Metformin on the Body via the Urine Proteome

**DOI:** 10.3390/biom15020241

**Published:** 2025-02-07

**Authors:** Yuzhen Chen, Haitong Wang, Minhui Yang, Ziyun Shen, Youhe Gao

**Affiliations:** Gene Engineering Drug and Biotechnology Beijing Key Laboratory, College of Life Sciences, Beijing Normal University, Beijing 100875, China; 202321200036@mail.bnu.edu.cn (Y.C.); 202221200042@mail.bnu.edu.cn (H.W.); 202221200019@mail.bnu.edu.cn (M.Y.); 202121200043@mail.bnu.edu.cn (Z.S.)

**Keywords:** urine, proteomics, post-translational modifications, metformin

## Abstract

Metformin is the first-line medication for treating type 2 diabetes mellitus, with more than 200 million patients taking it daily. Its effects are extensive and play a positive role in multiple areas. Can its effects and potential mechanisms be explored through the urine proteome? In this study, 166 differential proteins were identified following the administration of 150 mg/(kg·d) of metformin to rats for five consecutive days. These included complement component C6, pyruvate kinase, coagulation factor X, growth differentiation factor 15, carboxypeptidase A4, chymotrypsin-like elastase family member 1, and L-lactate dehydrogenase C chain. Several of these proteins have been reported to be directly affected by metformin or associated with its effects. Multiple biological pathways enriched by these differential proteins, or proteins containing differentially modified peptides, have been reported to be associated with metformin, such as the glutathione metabolic process, negative regulation of gluconeogenesis, and the renin–angiotensin system. Additionally, some significantly changed proteins and enriched biological pathways, not yet reported to be associated with metformin’s effects, may provide clues for exploring its potential mechanisms. In conclusion, the application of the urine proteome offers a comprehensive and systematic approach to exploring the effects of drugs, providing a new perspective on the study of metformin’s mechanisms.

## 1. Introduction

Metformin, the first-line medication for treating type 2 diabetes mellitus (T2DM), has been used clinically for over 60 years and is taken daily by more than 200 million T2DM patients worldwide [1]. In addition to lowering blood glucose, it has demonstrated benefits in cognitive function improvement [2], tumor suppression [3], cardiovascular protection [4], antiaging [5], and weight loss [6]. Despite its widespread use, the exact mechanisms of metformin remain incompletely understood.

Proteomics research reveals the composition and dynamics of proteins within cells or organisms by analyzing protein structure, expression, post-translational modifications, and protein interactions [7]. Drug-regulated post-translational modifications of proteins can serve as molecular target-binding markers, identifying pathways involved in drug regulation and helping to elucidate drug targets and mechanisms of action [8]. However, the effects of drugs on post-translational modifications have received little attention.

Urine, not strictly regulated by homeostatic mechanisms, can accommodate and accumulate more changes, reflecting changes in all organs and systems of the body earlier and more sensitively [9]. In addition, urinary proteins do not directly originate from drugs, with changes in the urine proteome reflecting the body’s overall response to the drugs. Various factors, such as age, genetics, gender, diet, and exercise, inevitably affect the urine proteome. Animal models, whose genetic and environmental factors can be controlled, are suitable choices [10].

**Figure 1:**
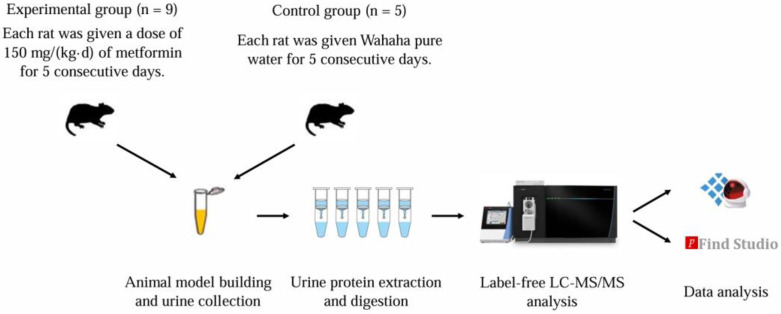
Technical route for exploring the effects of metformin on the rat urine proteome.

Therefore, considering metformin’s broad range of effects and its large patient base, can we leverage urine’s ability to comprehensively, systematically, and sensitively reflect the body’s state to explore metformin’s effects and potential mechanisms in greater detail? In this study, rat models were established using the intragastric administration of metformin to explore its effects on the body through the urine proteome. Exploring the known or unknown mechanisms of metformin provides a new perspective on the study of metformin’s mechanisms (as shown in Figure 1).

## 2. Materials and Methods

### 2.1. Urine Collection

Fourteen healthy male Sprague Dawley (SD) rats (200 ± 20 g) aged 6–7 weeks were purchased from Beijing Vital River Laboratory Animal Technology Co., Ltd., Beijing, China. The experiment began after the rats were acclimated in a standard environment [room temperature (22 ± 1) °C, humidity 65–70%] for 3 weeks, by which time the rats weighed 370 ± 30 g. Animal experiments were reviewed and approved by the Ethics Committee of the College of Life Sciences, Beijing Normal University (No. CLS-EAW-2020-034).

Metformin was dissolved in Wahaha pure water and administered intragastrically once a day at the same time for 5 days. The experimental group (*n* = 9) was given a dose of 150 mg/kg of metformin, which has been reported to achieve plasma concentrations of metformin in rats comparable to those in humans [11]. The control group (*n* = 5) was given Wahaha pure water. After 5 days of treatment, rats were uniformly placed in metabolic cages to collect urine samples for 12 h. The urine samples were then stored at −80 °C.

### 2.2. Urine Sample Preparation for Label-Free Analysis

The collected urine samples were centrifuged at 12,000× *g* for 40 min at 4 °C. The supernatant was transferred to a new centrifuge tube. Pre-cooled anhydrous ethanol, three times the volume of the supernatant, was added, mixed thoroughly, and then precipitated overnight at −20 °C. Following centrifugation at 12,000× *g* for 30 min at 4 °C, the supernatant was discarded. The protein precipitate was then suspended in an appropriate lysis buffer (8 mol/L urea, 2 mol/L thiourea, 25 mmol/L dithiothreitol, and 50 mmol/L Tris). After being fully dissolved, the sample was centrifuged again at 12,000× *g* for 30 min at 4 °C. The supernatant was transferred to the new centrifuge tube to obtain urine protein extract. Protein concentration was quantified using the Bradford kit assay (Applygen, Beijing, China).

A total of 100 μg of protein was digested with trypsin (Trypsin Gold, Promega, Madison, WI, USA) using filter-aided sample preparation (FASP) methods [12]. Detailed experimental procedures are available in the Supplementary Materials. The digested peptides were eluted from ultrafiltration membranes, desalted with HLB columns (Waters, Milford, MA, USA), dried in a vacuum desiccator, and stored at −80 °C.

### 2.3. Liquid Chromatography Coupled with Tandem Mass Spectrometry Analysis

The digested peptides were dissolved in 0.1% formic acid, and the peptide concentration was quantified using a BCA kit. The peptides were then diluted to a final concentration of 0.5 μg/μL. A mixed peptide sample was prepared by combining 14 μL from each sample, and separation was performed using a high pH reversed-phase peptide fractionation kit (Thermo Fisher Scientific, Rockford, IL, USA). Ten fractions were collected by centrifugation, dried in a vacuum desiccator, and redissolved in 0.1% formic acid. The indexed retention time (iRT) reagent (Biognosis, Schlieren, Switzerland) was then added at a ratio of 1:10 (iRT-to-sample). For analysis, 1 μg of the peptide from each sample was loaded and separated using the EASY-nLC 1200 system (Thermo Fisher Scientific, Waltham, MA, USA), and analysis was performed using an Orbitrap Fusion Lumos Tribrid Mass Spectrometer (Thermo Fisher Scientific, Waltham, MA, USA). The liquid chromatography coupled with tandem mass spectrometry (LC-MS/MS) settings are detailed in the Supplementary Materials.

### 2.4. Database Searching and Data Processing

Ten fractions obtained from the high pH reversed-phase peptide fractionation kit were analyzed by mass spectrometry in a data-dependent acquisition (DDA) mode to generate a spectral library. The DDA results were then imported into Proteome Discoverer software (version 2.1, Thermo Fisher Scientific) for searching against the *Rattus norvegicus* database using SEQUEST HT. The search results were used to establish the DIA method. The width and number of windows were calculated based on the *m*/*z* distribution density. Individual samples were analyzed using the data-independent acquisition (DIA) mode, with each sample run in triplicate. After every 8 runs, a single DIA analysis of the pooled peptides was performed to control the quality of the whole analytical process. The results were then imported into Spectronaut Pulsar (version 19, Biognosys AG, Schlieren, Switzerland) software for analysis and processing. The peptide intensity was calculated by summing the peak areas of the respective fragment ions for MS^2^, while protein intensity was calculated by summing the peptide intensities. All results were filtered with a *Q* value < 0.01 (corresponding to false discovery rate [FDR] < 1%), and only proteins containing at least two specific peptides were retained.

Modification information of the proteome was obtained using pFind Studio software (version 3.2.0, Institute of Computing Technology, Chinese Academy of Sciences, Beijing, China). Data collected by the Orbitrap Fusion Lumos Tribrid Mass Spectrometer were analyzed for label-free quantification, with original data files searched against the *Rattus norvegicus* UniProt canonical database (updated in September 2024). For the search, “HCD-FTMS” was selected for “MS Instrument”, “Trypsin_P KR P C” was chosen for trypsin digestion, and the maximum number of missed cleavages allowed per peptide was two. Both precursor and fragment tolerances were set to ±20 ppm. To identify global modifications, “Open Search” was selected. FDRs at the spectra, peptide, and protein levels were all kept below 1%. The number of peptide mass spectra in each sample was extracted from the analysis results in pFind Studio using the script “pFind_protein_contrast_script.py” [13,14].

### 2.5. Data Analysis

The proteins and the number of modified peptide mass spectra identified in the experimental and control groups were compared separately. Differential proteins and differentially modified peptides were screened with the following criteria: fold change (FC) ≥ 1.5 or ≤0.67, and *p* < 0.05 by two-tailed unpaired *t*-test analysis. Relaxed screening criteria for FC and *p*-values were applied to enhance sensitivity and detection rates, enabling the identification of a broader spectrum of potential biological changes while ensuring biological significance.

Hierarchical cluster analysis (HCA) and principal component analysis (PCA) were performed using the SRplot web server (http://www.bioinformatics.com.cn/ (accessed on 26 December 2024)). Functional enrichment analysis of differential proteins or proteins containing differentially modified peptides was performed using the Database for Annotation, Visualization, and Integrated Discovery (DAVID) (https://davidbioinformatics.nih.gov/ (accessed on 26 December 2024)) and Metascape (https://metascape.org/gp/ (accessed on 17 January 2025)) [15]. Functional analysis was further supported by searching the PubMed database (https://pubmed.ncbi.nlm.nih.gov (accessed on 26 December 2024)) for relevant studies in the literature.

## 3. Results and Discussion

### 3.1. Urine Proteome Analysis

#### 3.1.1. Identification of Urinary Proteins

Fourteen samples from both the experimental and control groups, collected after intragastric administration, were analyzed by LC-MS/MS. A total of 1542 proteins were identified based on the criteria that each protein contained at least two specific peptides and had an FDR < 1% at the protein level. The urinary proteins of the two groups were compared, and 166 differential proteins were identified under the screening conditions of FC  ≥  1.5 or  ≤0.67 and *p* < 0.05. Among these, 88 were down-regulated and 78 were up-regulated. Detailed information on these differential proteins is listed in Table S1.

**Figure 2:**
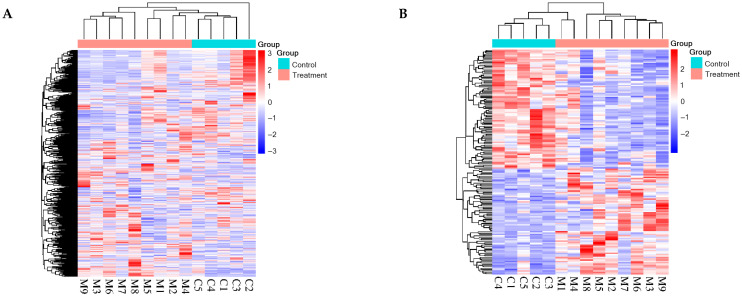
HCA of total and differential proteins distinguished the samples from the experimental and control groups (C1–C5: control group samples; M1–M9: experimental group samples): (**A**) total proteins; (**B**) differential proteins.

**Figure 3:**
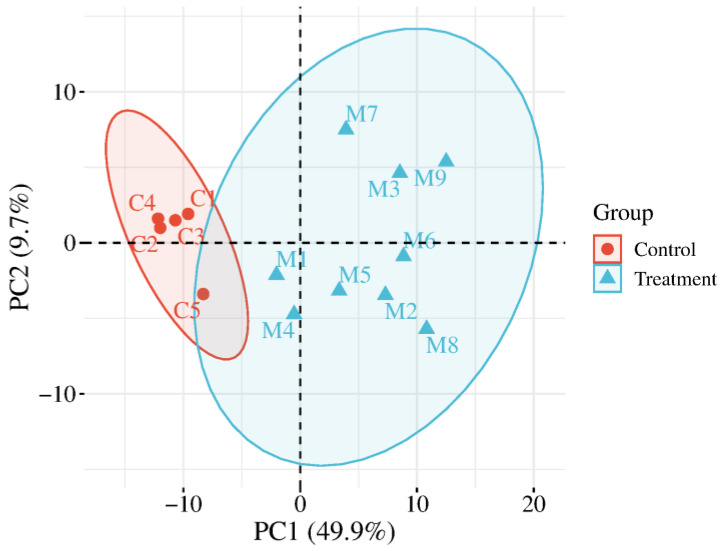
PCA of differential proteins distinguished the samples from the experimental and control groups (C1–C5: control group samples; M1–M9: experimental group samples).

HCA was performed on the identified total proteins and differential proteins, respectively (Figure 2), and PCA was performed on the differential proteins (Figure 3), both of which clearly distinguished the samples from the experimental and control groups.

#### 3.1.2. Randomized Grouping Test for Total Proteins

To assess the possibility of random generation of the identified differential proteins, a randomized grouping test was performed on the total proteins. Fourteen samples were randomly divided into two new groups, resulting in a total of 2002 combinations. These combinations were then screened for differences based on the same criteria (FC  ≥  1.5 or  ≤ 0.67, *p* < 0.05). The average number of differential proteins yielded was 38.46, indicating that at least 76.83% of the differential proteins were not randomly generated. This supports the reliability of the 166 differential proteins identified in this study.

#### 3.1.3. Analysis of Differential Proteins

Functional analysis of 166 differential proteins was performed using the PubMed database. Four of these proteins have been reported to be directly affected by metformin (Table 1). Other members of two protein families have also been reported to be directly affected by metformin (Table 2). Twenty-seven proteins have not been reported to be directly affected by metformin, but they are functionally associated with the efficacy of metformin (Table 3). Detailed information for each protein is as follows.

##### Differential Proteins Reported to Be Directly Affected by Metformin

(1) Pyruvate kinase

Metformin enhances the activity of pyruvate kinase in hepatocytes, inhibiting gluconeogenesis [16], which is one of the main ways metformin exerts its hypoglycemic effect [17]. Some researchers have proposed that metformin potentiates the allosteric activation of pyruvate kinase by fructose-1,6-diphosphate, suggesting pyruvate kinase as the locus of metformin’s clinical action [18].

In addition, pyruvate kinase M2 (PKM2), a downstream molecule in the PI3K/AKT/mTOR signaling pathway, is overexpressed in nearly all tumor cells and plays a crucial role in the Warburg effect [19,20]. Metformin has been shown to down-regulate PKM2 expression in gastric cancer cells [21], esophageal cancer cells [22], and breast cancer cells [23]. Modulating PKM2 activity or expression has been proposed as a promising strategy to enhance the anticancer effect of metformin.

(2) Growth differentiation factor 15 (GDF15)

Several studies have shown that metformin increases GDF15 levels [24,25,26,27]. GDF15 is a cytokine with anti-inflammatory properties that enhances insulin sensitivity, suppresses appetite, reduces body weight in both diabetic and non-diabetic patients, and improves the prognosis of diabetic patients. GDF15 levels are also associated with the progression of diabetic complications, including thrombosis, diabetic nephropathy, diabetic neuropathy degeneration, and diabetic retinopathy [28]. GDF15 has been identified as a novel biomarker for metformin administration in patients with abnormal blood glucose, and its concentration may reflect the dosage of metformin used [25]. In addition, due to its anti-inflammatory and appetite suppressant effects, GDF15 shows significant potential for treating various metabolic disorders such as obesity, T2DM, non-alcoholic fatty liver disease, cardiovascular disease, and cancer cachexia [29].

(3) Cystathionine gamma-lyase (CSE)

Metformin can alleviate atherosclerosis by regulating CSE expression and promoting hydrogen sulfide (H_2_S) production [30]. In rats exposed to bisphenol A (BPA), metformin up-regulates the expression of CSE and cystathionine β-synthase (CBS), reduces serum homocysteine levels, and protects against BPA-induced hepatic injury [31].

(4) Cytochrome P450

In rats, metformin is primarily metabolized by hepatic microsomal cytochrome P450 (CYP) isozymes, including CYP2C11, 2D1, and 3A1/2 [32].

##### Other Members of the Protein Family Reported to Be Directly Affected by Metformin

(1) Solute carrier family 22, member 21

Solute carrier family 22 is an organic cation transporter protein transporting various endogenous and exogenous substances. The main transporter proteins for metformin are solute carrier family 22 member 1 (OCT1) and member 4 (OCTN1) [33]. Among these, genetic polymorphisms in OCT1 can affect the pharmacokinetics of metformin and gastrointestinal intolerance, influencing individual responses to metformin [34,35,36].

(2) Carboxypeptidase A4

Carboxypeptidase A4 (FC = 49.79, *p* = 8.39 × 10^−3^) exhibited the highest FC value among the 166 differential proteins identified in this study. A genetic variation in carboxypeptidase A6 (CPA6) has been reported to be associated with metformin response in patients with T2DM [37].

##### Newly Identified Proteins with Functions Associated with Metformin Efficacy

(1) Secreted Ly6/Plaur domain containing 2 (SLURP-2)

SLURP-2 (FC = 1.54, *p* = 5.67 × 10^−4^) had the fifth smallest *p*-value among all the differential proteins identified in this study. SLURP-2 is a new member of the Ly-6 superfamily, whose expression is up-regulated in psoriasis patients. It may be involved in the pathophysiological process of psoriasis through keratinocyte proliferation and T-cell differentiation/activation [38]. Several studies have shown that metformin improves treatment outcomes and metabolic syndrome in psoriasis patients [39,40,41], and long-term metformin use is associated with a reduced risk of developing psoriasis [42].

(2) Chymotrypsin-like elastase family member 1

Chymotrypsin-like elastase family member 1 (FC = 25.11, *p* = 3.91 × 10^−2^) exhibited the second-highest FC value among all the differential proteins identified in this study. The gene encoding this protein, *Cela1*, is expressed during lung development and is closely associated with the stretch-dependent remodeling of the lungs, both physiologically and pathologically. Its expression is increased in mice and humans with α-1 antitrypsin-deficient emphysema, making it a potential target for treating this condition [43]. Metformin has shown promise in treating emphysema in both mice and humans, particularly in slowing disease progression [44].

(3) BPI fold-containing family A member 2 (BPIFA2)

BPIFA2 levels are higher in the blood and urine of patients with acute kidney injury compared to those of healthy individuals, making it a potential early biomarker for this condition [45]. Even low doses of metformin exacerbate renal ischemia–reperfusion-induced acute kidney injury and increase mortality in mice [46].

(4) T-complex protein 1 subunit beta (TCP-1β)

TCP-1β can be used as a biomarker for the glomerular hyperfiltration phase of type 2 diabetic nephropathy [47]. Several studies suggest that metformin plays an important role in alleviating diabetic nephropathy [48,49,50].

(5) Nicotinate phosphoribosyltransferase

Nicotinate phosphoribosyltransferase is crucial in the metabolism of trigonelline, which is involved in synthesizing nicotinamide adenine dinucleotide (NAD+). It enhances mitochondrial activity and has great potential for ameliorating age-related muscle decline [51].

(6) Coagulation factor X and XII

Diabetic patients exhibit a hypercoagulable state compared to healthy individuals, characterized by high levels of coagulation factors (II, V, VII, VIII, and X) and low levels of anticoagulants (protein C) [52].

(7) Calpain-2 catalytic subunit

Metformin has been shown to reduce cardiovascular disease mortality, all-cause mortality, and the risk of cardiovascular events in patients with coronary artery disease [93]. In patients with T2DM, treatment with metformin is associated with reduced cardiovascular mortality and morbidity [4]. It also protects the heart against hypertrophic and apoptotic remodeling following myocardial infarction [94]. Calpain 2 has been reported to be significantly elevated in atrial samples from patients with atrial fibrillation compared to those in sinus rhythm, suggesting its role in the development of atrial fibrillation in patients with heart valve disease and diabetes [53].

##### Differential Proteins Associated with High Blood Glucose or Diabetes

(1) Integrin subunit alpha V

Hyperglycemia reduces the expression of integrin subunits alpha v and alpha 5 on the surface of dermal fibroblasts, which affects fibroblast migration and the wound healing process. This may contribute to defective wound healing in diabetes [54].

(2) 5-oxoprolinase (OPLAH)

Down-regulation of OPLAH in human skeletal muscle cells (HSKMCs) may lead to insulin resistance and glucose uptake impairment through oxidative stress, making it a potential therapeutic target for T2DM [55].

(3) Triokinase/FMN cyclase

As a new NRF2 target gene, *Tkfc*, which encodes triokinase/FMN cyclase, is up-regulated in atypical NRF2 activation. It plays an important role in promoting hepatic fructose metabolism and gluconeogenesis, influencing blood glucose homeostasis [56].

(4) Regenerating family member 3 beta (Reg3β)

Recombinant Reg3β protein prevents streptozotocin-induced diabetes and pancreatic islet beta cell damage in mice [57].

(5) Lysozyme f1

Lysozyme structure and function are altered in patients with T2DM. L-lysine, a chemical molecular chaperone, significantly improves lysozyme structure and function, reverses glycosylation changes, increases lysozyme activity, and helps prevent diabetic complications [58].

(6) Arginase-1 (ARG1)

Arginase 1 (ARG1) and arginase 2 (ARG2) play important roles in regulating β-cell function, insulin resistance (IR), and vascular complications by regulating L-arginine metabolism, nitric oxide (NO) production, inflammatory responses, and oxidative stress. Abnormal alterations in arginase expression and activity are closely associated with the development of diabetes and its complications. Targeting arginase is a potential therapeutic approach for diabetes [59].

##### Differential Proteins Associated with Cancer

Cancer has become the leading cause of death among diabetic patients in high-income countries [95]. A meta-analysis of 10,695,875 patients with T2DM showed that those using metformin had a significantly lower risk of developing cancer compared to those using other glucose-lowering medications. Subgroup analyses showed a significant reduction in the risk of bladder cancer, colorectal cancer, gastric cancer, liver cancer, lung cancer, pancreatic cancer, and prostate cancer among metformin users [3]. Several other studies have demonstrated metformin’s potential in the treatment of glioma [86], cervical cancer [96], acute myeloid leukemia [97], breast cancer [98], and ovarian cancer [99]. The differential proteins associated with cancer are as follows:

(1) L-lactate dehydrogenase C chain, LDH-C

L-lactate is produced by the reduction of pyruvate catalyzed by lactate dehydrogenase. The level of lactate dehydrogenase 5 (LDH5) is higher in many tumor tissues compared to normal tissues. *LDHC* is also expressed in a variety of tumors, including lung cancer, melanoma, prostate cancer, and breast cancer [60,61]. Enzymes involved in L-lactate metabolism are closely related to the pathophysiological processes of diabetes, cancer, and other diseases, potentially offering new strategies for treatment [60].

(2) Mucosal pentraxin

Heme, which has been reported to be associated with red meat consumption and colon cancer risk, can lead to more than a 10-fold down-regulation of *Mptx* in rats [62]. *Mptx* may be involved in processing damaged cells in the colonic mucosa, and its expression is associated with colonic cell renewal. It could serve as a marker for diet-induced stress in the colonic mucosa [62,63]. Meta-analysis studies have shown that metformin use is associated with a reduced risk of colon cancer [64], a significant decrease in overall colon cancer mortality, and a better prognosis for colon cancer patients [65,66].

(3) Dynein cytoplasmic 1 heavy chain 1 (DYNC1H1)

DYNC1H1 encodes the cytoplasmic dynein heavy chain family, which links phagocytosis to apoptosis, preventing various diseases, including cancer, neurodegenerative diseases, and autoimmune diseases [67,68]. Studies suggest that DYNC1H1 is a new prognostic biomarker for hepatocellular carcinoma, associated with the epithelial–mesenchymal transition and immune infiltration, and holds great potential for early diagnosis and effective intervention in hepatocellular carcinoma [67]. Metformin may influence the early progression of hepatocellular carcinoma associated with non-alcoholic fatty liver disease/non-alcoholic steatohepatitis (NAFLD/NASH) by modulating macrophage polarization and T-cell infiltration [69].

(4) Phosphatidylinositol transfer protein alpha isoform (PITP-alpha)

PITP is an abundant and ubiquitous soluble protein. Increased expression of PITPα/β in gastric cancer tissues is associated with poor prognosis of gastric cancer, making it a potential target for cancer treatment [70].

In addition, decreased expression of PITPα is related to the pathological improvement of Duchenne muscular dystrophy and is a potential target for its treatment [71]. Metformin improves muscle function and reduces neuromuscular deficits in mice with muscular dystrophy, suggesting its therapeutic potential for Duchenne muscular dystrophy patients [72].

(5) Sodium-coupled monocarboxylate transporter 1

*SLC5A8*, the gene encoding sodium-coupled monocarboxylate transporter 1, functions as a tumor suppressor gene in various cancers. In cervical cancer, *SLC5A8* is silenced by DNA hypermethylation and histone deacetylation, serving as both a biomarker and a therapeutic target for diagnosis and prognosis [73]. *SLC5A8* has also been identified as an oncogene in colorectal cancer, where it undergoes methylation and silencing [74]. Among the hypermethylated and silenced genes associated with acute myeloid leukemia in mixed-lineage leukemia partial tandem duplication (*MLL*-PTD), silencing of the tumor suppressor gene *SLC5A8* promotes leukemogenesis [75]. In mouse breast cancer, *SLC5A8* is silenced, and reactivating its expression may offer a new therapeutic strategy [76].

(6) Epidermal growth factor receptor kinase substrate 8 (Eps8)

Eps8 is highly expressed in various human tumors, including colorectal cancer [77], pituitary tumors [78], oral squamous cell carcinoma [79], esophageal cancer [80], pancreatic cancer [81], and cervical cancer [82]. It participates in many signaling pathways associated with carcinogenesis, metastasis, and proliferation and is a biomarker of poor prognosis in cancer patients.

(7) Alpha-N-acetylgalactosaminidase

Down-regulation of α-N-acetylgalactosaminidase expression mediated by shRNA inhibits the migration and invasion of breast and ovarian cancer cell lines. α-N-acetylgalactosaminidase is expected to be a potential anticancer therapeutic target [83].

(8) Annexin A8

Annexin A8 is overexpressed in pancreatic cancer [84], and its increased expression is associated with poor prognosis in early-stage pancreatic cancer. It may serve as a prognostic marker and a potential therapeutic target for pancreatic cancer [85].

(9) Ferritin light chain 1 (FTL)

Hypoxia-induced FTL regulates the epithelial–mesenchymal transition and can serve as a prognostic marker for glioma. It is also a novel biomarker for the response to the anti-tumor drug temozolomide [87]. Metformin inhibits glioma cell stemness and epithelial–mesenchymal transitions by modulating the activity of YAP, a key effector in the Hippo pathway [86].

(10) Beta-glucuronidase

High levels of urinary beta-glucuronidase are observed in patients with bladder cancer [88].

##### Differential Proteins Associated with Cognitive Dysfunction

Cognitive dysfunction has also been reported as one of the many complications of diabetes mellitus [100]. Several studies have shown that diabetic patients are at an increased risk of developing dementia, including Alzheimer’s disease [101,102,103,104]. Metformin plays a positive role in ameliorating cognitive impairment and alleviating memory loss [2,105,106,107]. The differential proteins associated with cognitive dysfunction are as follows:

(1) Secernin-2

*SCRN2*, *LCMT1*, *LRRC46*, *MRPL10*, *SP6*, and *OSBPL7* are significantly associated with the Aβ standardized uptake value ratio in the brain. Single nucleotide polymorphisms (SNPs) in these genes are also associated with reduced hippocampal volume and cognitive scores. These genes may be new therapeutic targets for Alzheimer’s disease [89].

(2) Vitronectin (VTN)

VTN is a multifunctional glycoprotein. Both VTN and its receptors have been associated with various diseases, including tumors, coagulation disorders, inflammatory diseases, and neurodegenerative disorders. VTN plays an important role in neuronal function and neurodegenerative diseases. It is involved in neural differentiation, neuro-nutrition, and neurogenesis, regulating axon size and supporting and guiding neuronal extension. Additionally, VTN interacts with integrin receptors in vascular endothelial cells to reduce the permeability of the blood–brain barrier, thus protecting the brain [90].

(3) Complement component C6

Complement component C6 (FC = 1.96, *p* = 2.66 × 10^−5^) has the smallest *p*-value among all differential proteins identified in this study. Complement is an important factor in the progression of neurodegenerative diseases such as Alzheimer’s disease, amyotrophic lateral sclerosis, and schizophrenia [91]. Activation of the innate immune response, particularly through the complement system’s terminal pathway, can lead to the formation of the membrane attack complex (MAC) and delay peripheral nervous system regeneration, causing neuronal damage. Complement component C6 is important in activating the complement system and MAC formation. And C6 deficiency facilitates neuronal recovery after trauma [92].

#### 3.1.4. Biological Pathway Analysis

**Figure 4:**
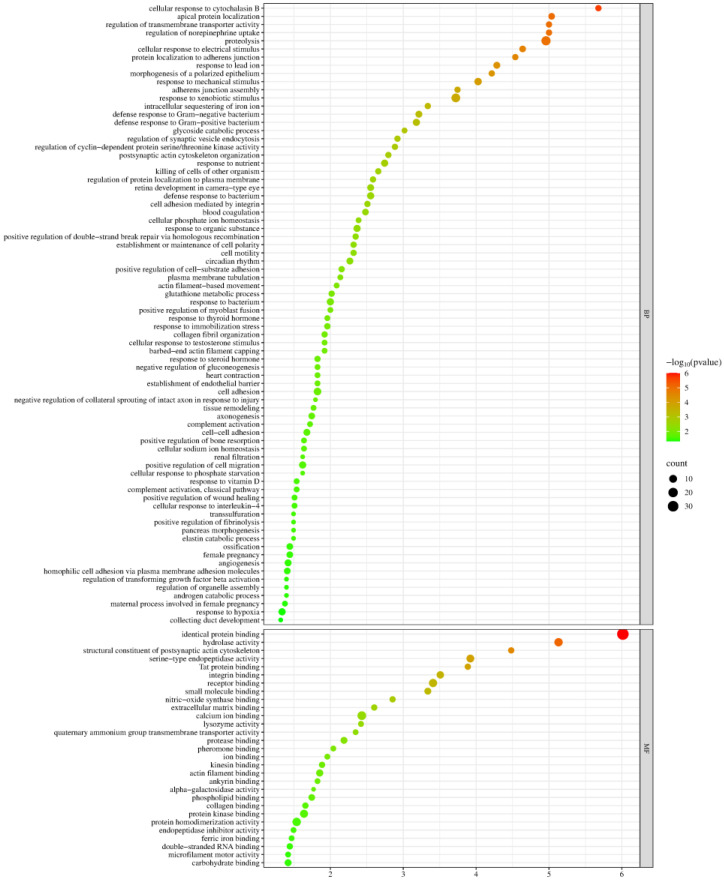
Enrichment analysis of biological processes and molecular functions of identified differential proteins (*p* < 0.05). The *X*-axis represents the *p*-values (−log 10) in the annotation categories. The *Y*-axis represents biological processes (BPs) and molecular functions (MFs).

Biological process and molecular function enrichment analyses of the identified differential proteins were performed using the DAVID database (Figure 4).

These differential proteins were mainly involved in biological processes such as cellular response to cytochalasin B, apical protein localization, regulation of norepinephrine uptake, regulation of transmembrane transporter activity, proteolysis, glycoside catabolic process, regulation of cyclin-dependent protein serine/threonine kinase activity, blood coagulation, glutathione metabolic process, establishment of endothelial barrier, and negative regulation of gluconeogenesis. Among these, blood coagulation, glutathione metabolic process, establishment of endothelial barrier, and negative regulation of gluconeogenesis have been reported to be associated with metformin action. Diabetic patients exhibit hypercoagulability compared to healthy individuals [52]. Metformin modulates glutathione metabolism and influences the progression of thyroid cancer [108]. Vascular endothelium plays a crucial role in regulating cardiovascular function, and several studies have shown that metformin improves endothelial function [109,110,111,112]. The inhibition of gluconeogenesis is one of the main pathways through which metformin exerts its hypoglycemic effect [17].

Among the molecular functions, most of these differential proteins were found to have functions including identical protein binding, hydrolase activity, structural constituent of the postsynaptic actin cytoskeleton, serine-type endopeptidase activity, Tat protein binding, integrin binding, receptor binding, small molecule binding, nitric-oxide-synthase binding, and lysozyme activity. Studies have shown that nitric oxide synthase is important in oxidative stress and vascular diseases [113]. The uncoupling of endothelial nitric oxide synthase (eNOS) may partially explain the pathogenesis of diabetic vascular diseases caused by decreased levels and impaired function of endothelial progenitor cells [114]. In addition, metformin promotes vascular regeneration and neurological recovery after spinal cord injury in aged mice by activating the AMPK/eNOS signaling pathway [115]. In patients with T2DM, the structure and function of lysozymes are altered. Reversing the changes caused by glycosylation and increasing lysozyme activity contribute to preventing diabetic complications [58].

**Figure 5:**
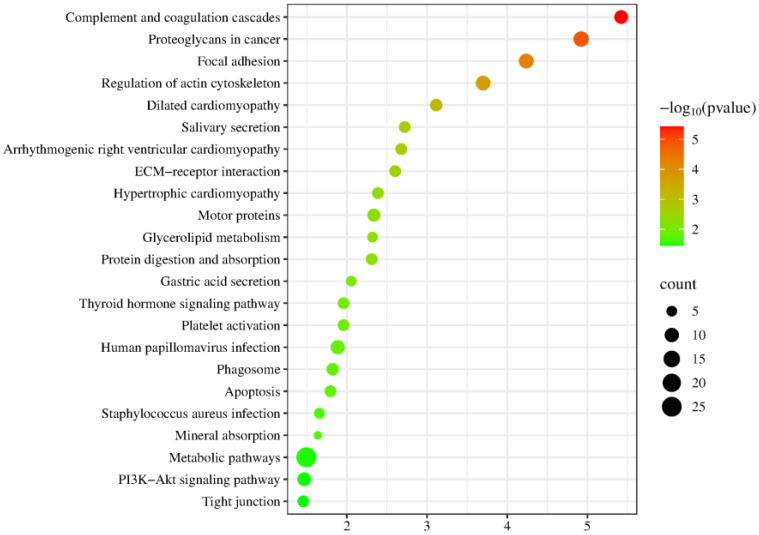
Enrichment analysis of the KEGG pathways of identified differential proteins (*p* < 0.05). The *X*-axis represents the *p*-values (−log 10) in the annotation categories, and the *Y*-axis represents the KEGG pathway.

Kyoto Encyclopedia of Genes and Genomes (KEGG) enrichment analysis revealed significant enrichment in pathways including complement and coagulation cascades, proteoglycans in cancer, focal adhesion, regulation of actin cytoskeleton, dilated cardiomyopathy, arrhythmogenic right ventricular cardiomyopathy, ECM–receptor interaction, hypertrophic cardiomyopathy, glycerolipid metabolism, platelet activation, and PI3K-Akt signaling pathway (Figure 5). Among these, glycerolipid metabolism, platelet activation, and PI3K-Akt signaling pathways have been reported to be associated with metformin efficacy. T2DM induces the inactivation of the glycerolipid metabolism pathway, and vanillin, which has antidiabetic properties, significantly reverses this change [116]. Platelet surface receptors and activation markers in T2DM patients differ significantly from those in healthy individuals [117]. Platelet activation has also been associated with the development of chronic diseases such as atherosclerosis, coronary artery disease, and cerebrovascular disease [118]. The PI3K-Akt signaling pathway is the most frequently activated in cancer. Under physiological conditions, this pathway is activated by insulin, growth factors, and cytokines, and it regulates key metabolic processes such as glucose metabolism and macromolecular biosynthesis, maintaining systemic metabolic homeostasis [119].

**Figure 6:**
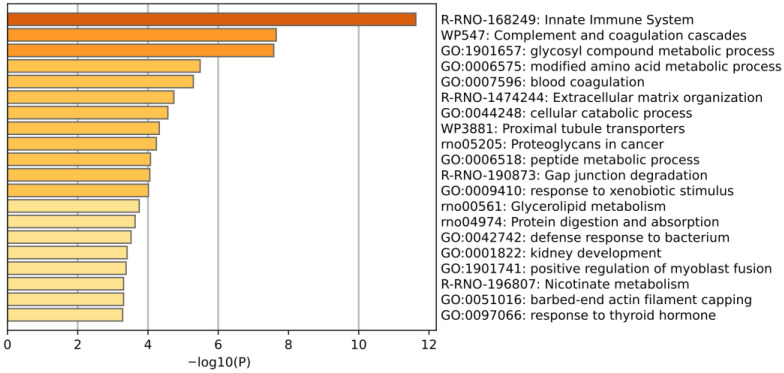
Enrichment analysis of identified differential proteins. The *X*-axis represents the *p*-values (−log 10) in the annotation categories, and the *Y*-axis represents the enriched items.

Using the Metascape database, several pathways or processes were significantly enriched, including the innate immune system, complement and coagulation cascades, the glycosyl compound metabolic process, the modified amino acid metabolic process, blood coagulation, and glycerolipid metabolism (Figure 6).

### 3.2. Urine Proteome Modification Analysis

#### 3.2.1. Identification of Differentially Modified Peptides

Using a non-labeled quantitative proteome method, data from 14 samples were obtained by LC-MS/MS analysis. A search based on open-pFind yielded detailed information on the mass spectra number of modified peptides in each sample, including the proteins they were located in and the types of modifications. Modified peptides with over 50% reproducibility were screened separately in the experimental and control groups, and the union set was created. A total of 3206 modified peptides were identified, with 285 differentially modified peptides selected based on the screening criteria of FC  ≥  1.5 or  ≤ 0.67 and *p* < 0.05. Detailed information is listed in Table S2, including peptide sequences, modification types, and the proteins in which the differentially modified peptides are located.

**Figure 7:**
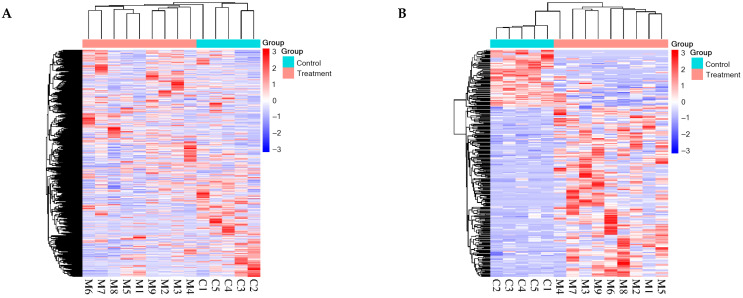
HCA of total and differentially modified peptides distinguished the samples from the experimental and control groups (C1–C5: control group samples; M1–M9: experimental group samples): (**A**) total modified peptides; (**B**) differentially modified peptides.

**Figure 8:**
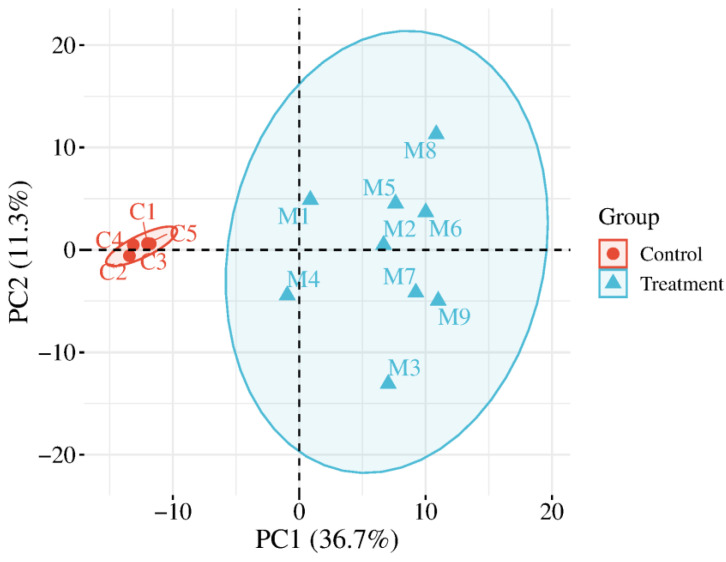
PCA of differentially modified peptides distinguished the samples from the experimental and control groups well (C1–C5: control group samples; M1–M9: experimental group samples).

HCA was performed on the identified total modified peptides and differentially modified peptides, respectively (Figure 7), and PCA was performed on the differentially modified peptides (Figure 8), both of which distinguished the samples from the experimental and control groups. The results of PCA (Figure 3 and Figure 8) for the differential proteins and the differentially modified peptides showed that sample points in the experimental group were more dispersed than those in the control group, indicating some inter-individual variation. It has been reported that SNPs in the genes encoding metformin transporters, such as OCT1, OCT2, MATE1, and MATE2, are significantly associated with the efficacy and toxicity of metformin [120]. In addition, individual response to metformin treatment is influenced by factors such as DNA methylation levels [121], individual health status [122,123], and gender [124]. This suggests the importance of considering individual differences in drug research.

#### 3.2.2. Randomized Grouping Test for Total Modified Peptides

To assess the possibility of the random generation of the identified differentially modified peptides, a randomized grouping test was performed on the total modified peptides. Fourteen samples were randomly divided into two new groups, resulting in a total of 2002 combinations. These combinations were then screened for differences based on the same criteria (FC  ≥  1.5 or  ≤ 0.67, *p* < 0.05). The average number of differentially modified peptides yielded was 132.8, indicating that at least 53.4% of the differentially modified peptides were not randomly generated.

#### 3.2.3. Analysis of Biological Pathways Enriched in Proteins Containing Differentially Modified Peptides

**Figure 9:**
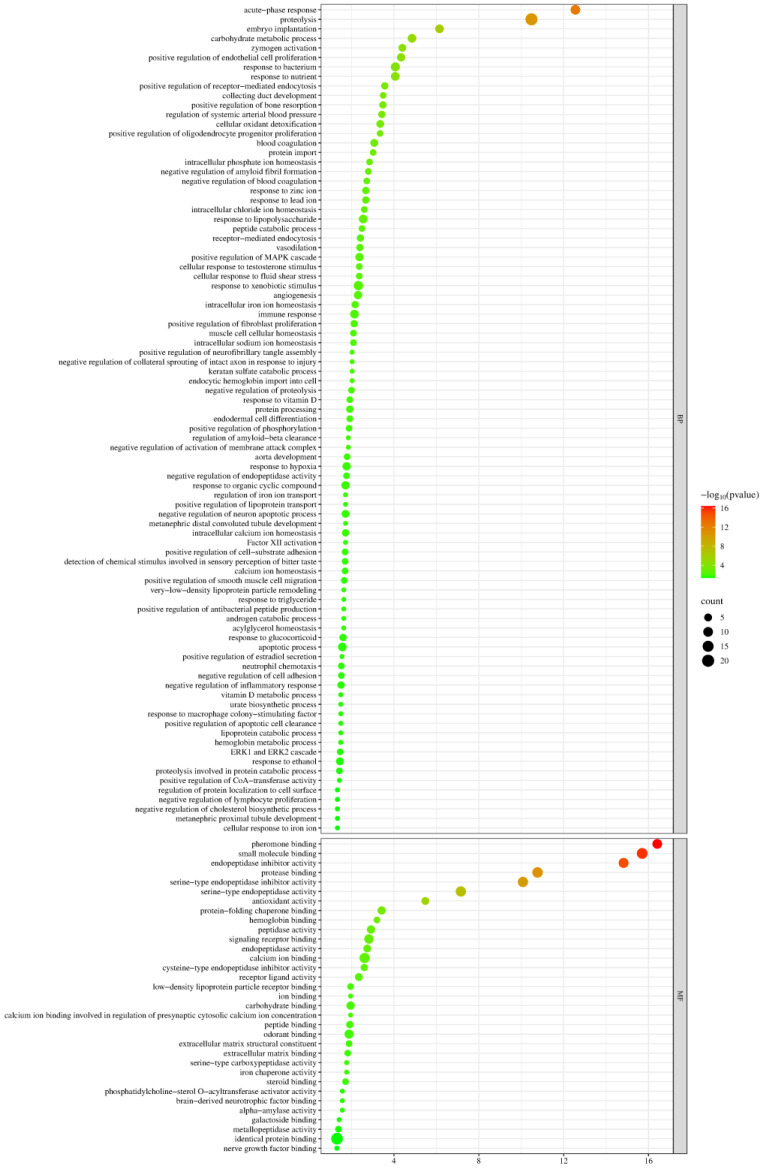
Enrichment analysis of biological processes and molecular functions of proteins containing differentially modified peptides (*p* < 0.05). The *X*-axis represents the *p*-values (−log 10) in the annotation categories, and the *Y*-axis represents biological processes (BPs) and molecular functions (MFs).

A total of 127 proteins containing differentially modified peptides were screened, with details provided in Table S3. Biological process and molecular function enrichment analyses of these proteins were performed using the DAVID database (Figure 9).

Proteins containing differentially modified peptides were mainly involved in biological processes such as the acute-phase response, proteolysis, carbohydrate metabolic process, zymogen activation, positive regulation of endothelial cell proliferation, response to bacterium, response to nutrients, positive regulation of receptor-mediated endocytosis, regulation of systemic arterial blood pressure, cellular oxidant detoxification, positive regulation of oligodendrocyte progenitor proliferation, blood coagulation, intracellular phosphate ion homeostasis, negative regulation of amyloid fibril formation, and negative regulation of blood coagulation.

Among the molecular functions, most of these proteins were found to have functions including pheromone binding, small molecule binding, endopeptidase inhibitor activity, protease binding, serine-type endopeptidase inhibitor activity, serine-type endopeptidase activity, antioxidant activity, protein-folding chaperone binding, and hemoglobin binding.

**Figure 10:**
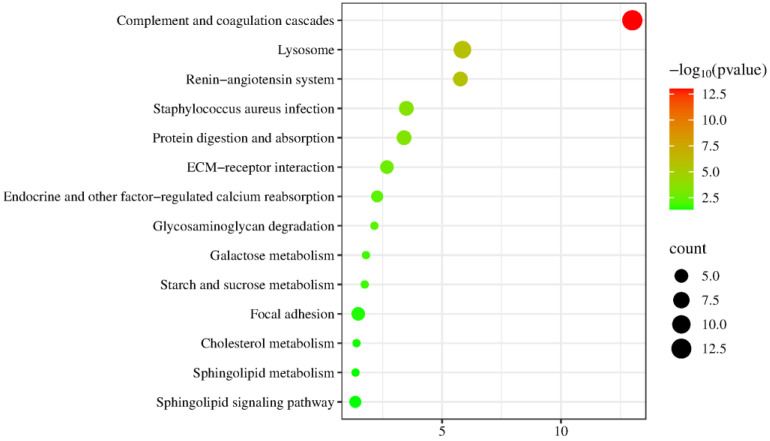
Enrichment analysis of the KEGG pathway of proteins containing differentially modified peptides (*p* < 0.05). The *X*-axis represents the *p*-values (−log 10) in the annotation categories, and the *Y*-axis represents the KEGG pathway.

KEGG enrichment analysis revealed significant enrichment in pathways including complement and coagulation cascades, lysosomes, the renin–angiotensin system, staphylococcus aureus infection, and protein digestion and absorption (Figure 10). Angiotensin II, a key component of the renin–angiotensin system (RAS), is a major target for effectively lowering blood pressure and preventing cardiovascular disease and kidney injury progression in diabetic patients [125].

**Figure 11:**
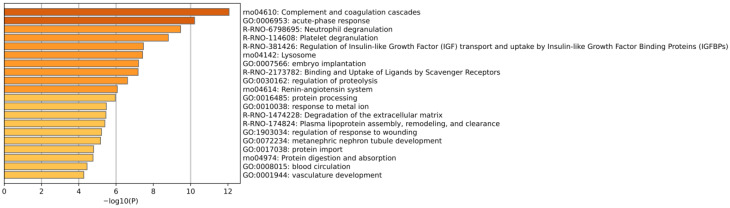
Enrichment analysis of proteins containing differentially modified peptides. The *X*-axis represents the *p*-values (−log 10) in the annotation categories, and the *Y*-axis represents the enriched items.

Using the Metascape database, several pathways or processes were significantly enriched, including complement and coagulation cascades, acute-phase response, neutrophil degranulation, platelet degranulation, regulation of insulin-like growth factor (IGF) transport and uptake by insulin-like growth factor binding proteins (IGFBPs), lysosomes, and the renin–angiotensin system (Figure 11).

#### 3.2.4. Differentially Modified Peptides from Presence to Absence or Absence to Presence

The differentially modified peptides with significant changes were screened based on the following criteria: peptides identified in more than half of the control group samples but not in the experimental group, or vice versa. A total of 100 such peptides were screened, with 9 identified in over half of the control group samples but not in the experimental group, and 91 identified in over half of the experimental group samples but not in the control group. Detailed information is listed in Table S4, including peptide sequences, modification types, the proteins in which the peptides are located, and the number of the modified peptide mass spectra for each sample.

**Figure 12:**
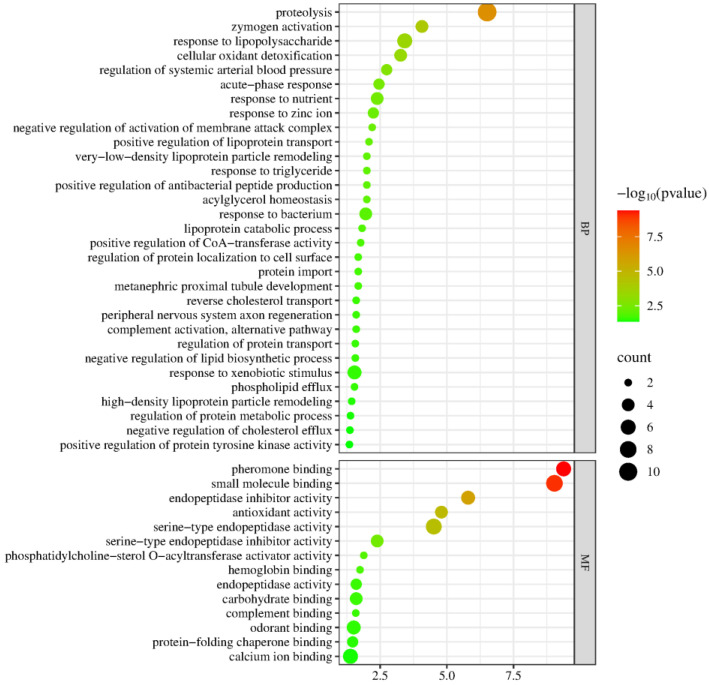
Enrichment analysis of biological processes and molecular functions of proteins containing differentially modified peptides, with changes from presence to absence or absence to presence (*p* < 0.05). The *X*-axis represents the *p*-values (−log 10) in the annotation categories, and the *Y*-axis represents biological processes (BPs) and molecular functions (MFs).

A total of 57 proteins containing differentially modified peptides from presence to absence or absence to presence were screened, with details provided in Table S5. Biological process and molecular function enrichment analyses of these proteins were performed using the DAVID database (Figure 12). These proteins were mainly involved in biological processes such as proteolysis, zymogen activation, response to lipopolysaccharide, cellular oxidant detoxification, regulation of systemic arterial blood pressure, acute-phase response, response to nutrients, negative regulation of activation of membrane attack complex, positive regulation of lipoprotein transport, acylglycerol homeostasis, very-low-density lipoprotein particle remodeling, response to triglycerides, lipoprotein catabolic process, positive regulation of CoA-transferase activity, reverse cholesterol transport, peripheral nervous system axon regeneration, negative regulation of lipid biosynthetic process, and high-density lipoprotein particle remodeling. Long-term use of metformin has been reported to reduce cholesterol and low-density lipoprotein levels in mice [126]. Among the molecular functions, most of these proteins were found to have functions including pheromone binding, small molecule binding, endopeptidase inhibitor activity, antioxidant activity, serine-type endopeptidase activity, serine-type endopeptidase inhibitor activity, phosphatidylcholine-sterol O-acyltransferase activator activity, hemoglobin binding, and carbohydrate binding.

**Figure 13:**
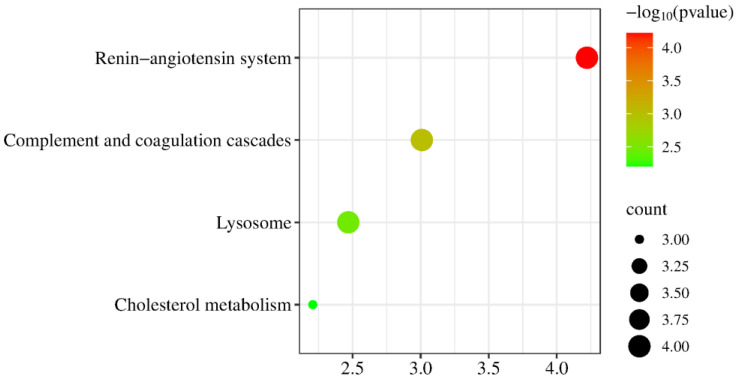
Enrichment analysis of the KEGG pathway of proteins containing differentially modified peptides from presence to absence or absence to presence (*p* < 0.05). The *X*-axis represents the *p*-values (−log 10) in the annotation categories, and the *Y*-axis represents the KEGG pathway.

KEGG enrichment analysis revealed significant enrichment in pathways including the renin–angiotensin system, complement and coagulation cascades, lysosomes, and cholesterol metabolism (Figure 13).

**Figure 14:**
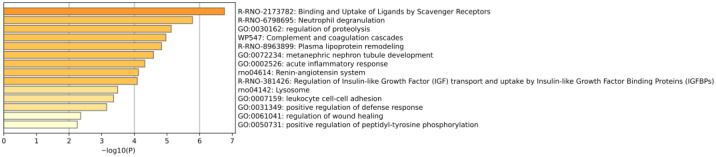
Enrichment analysis of proteins containing differentially modified peptides from presence to absence or absence to presence. The *X*-axis represents the *p*-values (−log 10) in the annotation categories, and the *Y*-axis represents the enriched items.

Using the Metascape database, several pathways or processes were significantly enriched, including binding and uptake of ligands by scavenger receptors, neutrophil degranulation, regulation of proteolysis, complement and coagulation cascades, plasma lipoprotein remodeling, the renin–angiotensin system, regulation of insulin-like growth factor (IGF) transport and uptake by insulin-like growth factor binding proteins (IGFBPs), and lysosomes (Figure 14).

This preliminary study was based on animal models. The differential proteins and differentially modified peptides, which are either associated with metformin or show significant changes, provide insights for exploring the mechanisms of metformin. More human urine samples should be included in future studies for further verification, which may provide new clues for studying both the known and unknown effects of metformin. In addition, this study only provides results based on comparisons of changes in the urine proteome after metformin administration. Further exploration is needed to understand how these differential proteins and differentially modified peptides specifically regulate related biological processes at the cellular and molecular levels. The mechanisms and specific processes of metformin in vivo also require further investigation.

## 4. Conclusions

After administering 150 mg/(kg·d) of metformin to rats for 5 consecutive days, multiple differential proteins reported to be directly affected by metformin or associated with its efficacy were identified. For example, pyruvate kinase is suggested as the locus of metformin’s clinical action, and GDF15 is a biomarker associated with metformin treatment. Differential proteins or proteins containing differentially modified peptides were enriched in biological pathways reported to be associated with metformin, such as the glutathione metabolic process, the negative regulation of gluconeogenesis, and the renin–angiotensin system. Some significantly changed proteins and enriched biological pathways, not previously reported to be associated with metformin’s effects, may provide clues for studying its potential mechanisms.

In conclusion, the application of the urine proteome helps to explore the known or unknown effects of drugs comprehensively and systematically, providing a new perspective on the study of metformin’s mechanisms.

## Figures and Tables

**Table 1:** Differential proteins reported to be directly affected by metformin.

UniProt ID	Protein Name	Trend *	FC	*p*-Value	Ref.
A0A0H2UI07	Pyruvate kinase	↓	0.54	3.44 × 10^−5^	[16,17,18,19,20,21,22,23]
G3V8K5	Growth differentiation factor 15	↑	2.01	3.70 × 10^−4^	[24,25,26,27,28,29]
A0A0G2K3C5	Cystathionine gamma-lyase	↓	0.50	1.06 × 10^−3^	[30,31]
F1LMN1	Cytochrome P450	↑	13.22	1.52 × 10^−2^	[32]

* “↓” indicates down-regulation, and ”↑” indicates up-regulation of the protein.

**Table 2:** Other members of the family of differential proteins reported to be directly affected by metformin.

UniProt ID	Protein Name	Trend *	FC	*p*-Value	Ref.
A0A0G2JVF2	Solute carrier family 22	↓	0.24	4.66 × 10^−2^	[33,34,35,36]
D3ZHS5	Carboxypeptidase A4	↑	49.79	8.39 × 10^−3^	[37]

* “↓” indicates down-regulation, and ”↑” indicates up-regulation of the protein.

**Table 3:** Newly identified differential proteins with functions associated with metformin efficacy.

UniProt ID	Protein Name	Trend *	FC	*p*-Value	Effect/Associated with a Disease	Ref.
D3ZUR5	Secreted Ly6/Plaur domain containing 2	↑	1.54	5.67 × 10^−4^	psoriasis	[38,39,40,41,42]
A0A0G2JSI5	Chymotrypsin-like elastase family member 1	↑	25.11	3.91 × 10^−2^	emphysema	[43,44]
Q63471	BPI fold-containing family A member 2	↑	24.76	4.66 × 10^−3^	acute kidney injury	[45,46]
Q5XIM9	T-complex protein 1 subunit beta (TCP-1-beta)	↓	0.36	6.86 × 10^−4^	diabetic nephropathy	[47,48,49,50]
G3V709	Nicotinate phosphoribosyltransferase	↓	0.42	4.67 × 10^−3^	aging	[51]
A0A0H2UI19	Coagulation factor XII	↑	1.51	2.91 × 10^−3^	coagulation factor	[52]
A0A0H2UHR6	Coagulation factor X	↑	2.29	2.08 × 10^−4^	coagulation factor	[52]
Q07009	Calpain-2 catalytic subunit	↓	0.21	9.08 × 10^−4^	atrial fibrillation	[53]
A0A0G2JVZ6	Integrin subunit alpha V	↓	0.27	4.92 × 10^−2^	high blood glucose/diabetes	[54]
P97608	5-oxoprolinase	↓	0.27	2.41 × 10^−2^	high blood glucose/diabetes	[55]
Q4KLZ6	Triokinase/FMN cyclase	↓	0.61	2.45 × 10^−2^	high blood glucose/diabetes	[56]
A0A0H2UHE4	Regenerating family member 3 beta	↑	4.26	2.99 × 10^−2^	high blood glucose/diabetes	[57]
D4A0W2	Lysozyme f1	↑	19.23	3.68 × 10^−3^	high blood glucose/diabetes	[58]
P07824	Arginase-1	↑	19.52	1.61 × 10^−2^	high blood glucose/diabetes	[59]
P19629	L-lactate dehydrogenase C chain (LDH-C)	↓	0.08	2.37 × 10^−2^	cancer	[60,61]
Q6TA48	Mucosal pentraxin	↓	0.12	4.38 × 10^−2^	cancer	[62,63,64,65,66]
F1LRT9	Dynein cytoplasmic 1 heavy chain 1	↓	0.21	1.59 × 10^−3^	cancer	[67,68,69]
P16446	Phosphatidylinositol transfer protein alpha isoform	↓	0.23	3.48 × 10^−2^	cancer, Duchenne muscular dystrophy	[70,71,72]
D3Z9E5	Sodium-coupled monocarboxylate transporter 1	↓	0.36	5.73 × 10^−4^	cancer	[73,74,75,76]
F1M3L7	Epidermal growth factor receptor kinase substrate 8	↓	0.58	5.68 × 10^−4^	cancer	[77,78,79,80,81,82]
Q66H12	Alpha-N-acetylgalactosaminidase	↓	0.65	6.84 × 10^−4^	cancer	[83]
Q4FZU6	Annexin A8	↑	4.07	2.72 × 10^−2^	cancer	[84,85]
P02793	Ferritin light chain 1	↑	4.48	1.97 × 10^−2^	cancer	[86,87]
F1LQQ8	Beta-glucuronidase	↑	8.86	6.67 × 10^−3^	cancer	[88]
Q6AYR8	Secernin-2	↓	0.25	1.03 × 10^−2^	cognitive dysfunction	[89]
Q3KR94	Vitronectin	↑	1.53	5.99 × 10^−4^	cognitive dysfunction	[90]
F1M7F7	Complement component C6	↑	1.96	2.66 × 10^−5^	cognitive dysfunction	[91,92]

* “↓” indicates down-regulation, and ”↑” indicates up-regulation of the protein.

## Data Availability

The mass spectrometry proteomics data were deposited to the ProteomeXchange Consortium (http://proteomecentral.proteomexchange.org (accessed on 5 January 2025)) via the iProX partner repository (https://www.iprox.cn/ (accessed on 5 January 2025)). The project ID is IPX0010695000 which was published by “Yuzhen Chen”.

## Supplementary Materials

The following supporting information can be downloaded at https://www.mdpi.com/article/10.3390/biom15020241/s1: Table S1. Differential proteins identified by comparison of the experimental and control groups. Table S2. Differentially modified peptides identified by comparison of the experimental and control groups. Table S3. Proteins containing differentially modified peptides. Table S4. Differentially modified peptides with changes from presence to absence or absence to presence. Table S5. Proteins containing differentially modified peptides with changes from presence to absence or absence to presence. Table S6. Information on the DIA acquisition window.
