# Supplementary material for: Exploring the Effects of Metformin on the Body via the Urine Proteome
Source: Biomolecules. 2025 Feb 7;15(2):241. doi: 10.3390/biom15020241 (PMC11853151; doi:10.3390/biom15020241)
Supplement: Supplementary file 1 [file biomolecules-15-00241-s001.zip › Supplementary Methods.pdf]

Supplementary Methods

Urine Protein Digestion

A total of 100 μg of protein was added to a 1.5 mL centrifuge tube. 25 mmol/L NH_4HCO_3 solution was added to make a total volume of 200 μL. 20 mmol/L dithiothreitol solution (DTT, Sigma) was added, vortexed, and mixed well. The sample was heated in a metal bath at 97℃ for 10 min. After cooling to room temperature, 50 mmol/L iodoacetamide solution (IAA, Sigma) was added, vortexed, and mixed well, and reacted at room temperature without light for 40 min. 200 μL of UA solution (8 mol/L urea, 0.1 mol/L Tris-HCl, pH 8.5) was added to a 10 kD ultrafiltration tube (Pall, Port Washington, NY, USA) and centrifuged twice at 14 000 ×*g* for 5 min at 18℃. The treated protein sample was added and centrifuged at 14 000 ×*g* for 40 min at 18℃. 200 μL of UA solution was then added and centrifuged at 14 000 ×*g* for 40 min at 18℃, repeated once. 25 mmol/L NH_4HCO_3 solution was added and centrifuged at 14 000 ×*g* for 40 min at 18℃, repeated once. The samples were digested overnight at 37 ℃ with trypsin (enzyme-to-protein ratio of 1:50).

LC-MS/MS Analysis

For analysis, 1 μg of the peptide from each sample was loaded into a reversed-phase C18 trap column (75 μm × 2 cm, 3 μm) at a flow rate of 0.3 μL/min and separated with a reversed-phase analytical column (50 μm × 15 cm, 2 μm) with mobile phase A (0.1% formic acid) and mobile phase B (0.1% formic acid in 80% acetonitrile). It was eluted with a 90-min gradient as follows: 0 min, 4% phase B; 0 min–2 min, 6% phase B; 2 min–62 min, 22% phase B; 62 min–78 min, 35% phase B; 78 min–90 min, 90% phase B, and then analyzed with an Orbitrap Fusion Lumos Tribrid Mass Spectrometer. The LC settings for DIA mode were identical to those used in DDA mode.

To generate a spectral library, ten fractions obtained by centrifugation were analyzed by mass spectrometry in DDA mode. The spray voltage was set to 2.25 kV. A full MS scan was acquired within a 350–1550 *m*/*z* scan range with a resolution of 120 000. The MS/MS scan was acquired in Orbitrap mode with a resolution of 30 000. The HCD collision energy was set to 30%. The top 20 precursors were selected and subjected to a 30-second dynamic exclusion period.

Individual samples were analyzed using DIA mode. The variable isolation window with 39 windows was adopted (Table S6). The spray voltage was set to 1.9 kV. The full scan was acquired within a 350–1500 *m*/*z* scan range with a resolution of 60 000, and the DIA scan was acquired in Orbitrap mode with a resolution of 30 000. The HCD collision energy was set to 32%.
